# Data reduction in protein serial crystallography

**DOI:** 10.1107/S205225252400054X

**Published:** 2024-02-08

**Authors:** Marina Galchenkova, Alexandra Tolstikova, Bjarne Klopprogge, Janina Sprenger, Dominik Oberthuer, Wolfgang Brehm, Thomas A. White, Anton Barty, Henry N. Chapman, Oleksandr Yefanov

**Affiliations:** a Center for Free-Electron Laser Science CFEL, Deutsche Elektronen-Synchrotron DESY, Notkestr. 85, 22607 Hamburg, Germany; b Deutsches Elektronen-Synchrotron DESY, Notkestr. 85, 22607 Hamburg, Germany; c Department of Physics, Universität Hamburg, Luruper Chaussee 149, 22761 Hamburg, Germany; d Universität Hamburg, Luruper Chaussee 149, 22761 Hamburg, Germany; Brookhaven National Laboratory, USA

**Keywords:** protein serial crystallography, data reduction, data compression, data quality evaluation

## Abstract

Serial crystallography (SX) has become an established technique for protein structure determination, especially when dealing with small or radiation-sensitive crystals and investigating fast or irreversible protein dynamics. The advent of newly developed multi-megapixel X-ray area detectors, capable of capturing over 1000 images per second, has brought about substantial benefits. However, this advancement also entails a notable increase in the volume of collected data. Today, up to 2 PB of data per experiment could be easily obtained under efficient operating conditions. The combined costs associated with storing data from multiple experiments provide a compelling incentive to develop strategies that effectively reduce the amount of data stored on disk while maintaining the quality of scientific outcomes. Lossless data-compression methods are designed to preserve the information content of the data but often struggle to achieve a high compression ratio when applied to experimental data that contain noise. Conversely, lossy compression methods offer the potential to greatly reduce the data volume. Nonetheless, it is vital to thoroughly assess the impact of data quality and scientific outcomes when employing lossy compression, as it inherently involves discarding information. The evaluation of lossy compression effects on data requires proper data quality metrics. In our research, we assess various approaches for both lossless and lossy compression techniques applied to SX data, and equally importantly, we describe metrics suitable for evaluating SX data quality.

## Abstract 2

Various approaches for lossless and lossy compression are evaluated, and suitable quality assessment metrics for serial crystallographic data – used in combination with lossy data reduction – are described.

## 1. Introduction

Serial crystallography (SX) has emerged as a standard tool for studying small or radiation-sensitive crystals and investigating protein dynamics in recent years (Chapman *et al.*, 2011 ▸; Boutet *et al.*, 2012 ▸; Chapman, 2019 ▸). This progress has been facilitated by the advancements in X-ray sources, including X-ray free-electron lasers (FELs) and fourth-generation synchrotrons. These modern X-ray sources generate high-intensity and coherent X-ray beams, coupled with improved focusing optics that enhance the flux density at the sample. As a result, the exposure time needed to capture measurable signals has significantly decreased.

Equally significant has been the concurrent advancement in detector technology. Modern detectors, such as EIGER (Dinapoli *et al.*, 2011 ▸), JUNGFRAU (Mozzanica *et al.*, 2018 ▸), Lambda (Pennicard *et al.*, 2013 ▸), ePix (Dragone *et al.*, 2013 ▸), AGIPD (Henrich *et al.*, 2011 ▸), LPD (Hart *et al.*, 2012 ▸) or DSSC (Porro *et al.*, 2010 ▸), have the capability to capture thousands of images per second. The development of these detectors, coupled with the aforementioned high-intensity photon sources, enables the collection of valuable images at a kilohertz frame rate (Tolstikova *et al.*, 2019 ▸). Combined with the tiling of detector modules to increase the number of pixels (up to 16 million pixels for EIGER or JUNGFRAU), this leads to very high overall data rates (Leonarski *et al.*, 2020 ▸). As an example, the EIGER2 XE 16M detector, developed by Dectris for synchrotron facilities, generates 16 megapixel images at a frame rate of 400 images per second. When uncompressed, the user gets a staggering data rate of 13.5 GB s^−1^. Considering the continuous operation typical in serial crystallography (SX), this amounts to approximately 1 PB per day of data. In the case of XFEL facilities, each of the two JUNGFRAU 16M detectors installed at SwissFEL can operate at a remarkable 2 kHz, generating data rates of up to 60 GB s^−1^. This equates to a potential accumulation of close to 4 PB per day by each detector. However, SwissFEL cannot be operated at such an extreme speed, which alleviates the burden on data storage systems. New detectors like ePixHR at LCLS2 and AGIPD 4M at European XFEL are expected to generate data at similar rates. Though it is technically feasible to save such data streams, the cumulative cost of doing so imposes a substantial burden on operational budgets. As a result, there is a compelling motivation to explore data-reduction strategies that preserve data while ensuring the quality of scientific outcomes remains unaffected.

Data reduction is a comprehensive concept that encompasses a range of techniques designed to decrease the size or complexity of a dataset while retaining essential information. These methods can include data compression, summarization, filtering, feature selection and dimensionality reduction. Often data-reduction strategies are divided into two big subgroups such as lossless and lossy algorithms.

In order to efficiently apply any data-reduction method, it is crucial to understand the data being processed. A typical diffraction pattern in SX comprises bright and sharply defined Bragg peaks, which originate from the studied crystals, and a relatively smooth background that arises from various factors, such as the sample delivery medium, disordered structure and solvent within the crystal, and parasitic scattering from the beamline. The intensities observed in various regions of the diffraction pattern can vary significantly, often differing by several orders of magnitude. Additionally, the useful signal represented by the Bragg peaks at high scattering angles may be comparable to the background noise. These features of diffraction patterns in SX affect the applicability of different compression algorithms.

Lossless compression techniques are frequently employed to reduce the size of scientific data, particularly when the signals recorded in each pixel of the detector are mostly zero or constant. However, constant signals are rarely observed in typical SX diffraction patterns. As a consequence, the effectiveness of lossless compression schemes is significantly diminished in this particular case. On the other hand, applying standard image compression techniques directly to SX data is challenging due to the significant signal variation observed in neighboring pixels, particularly near Bragg peaks. Thus, to achieve efficient data compression in SX, alternative compression approaches that are specifically designed to handle the sparse nature, high dynamic range, high noise level and sharp intensity changes observed in diffraction patterns are needed.

Our focus here is to evaluate different lossless and lossy data-compression methods and determine the appropriate metrics for evaluating the impact of lossy compression on the final SX data quality. Our research found that an effective strategy for data reduction in the case of strongly diffracting crystals is to selectively save only the images that exhibit a substantial number of Bragg peaks. Certainly, it is imperative to preserve all metadata necessary for subsequent processing of the condensed dataset (Bernstein *et al.*, 2020 ▸). As described below, this approach demonstrates remarkable efficacy, even when reprocessing previously collected data using new algorithms. Furthermore, our analysis shows that a non-linear reduction in the precision of the measured diffraction pattern intensities is the second most effective strategy for achieving lossy compression. Moreover, we show that binning, which effectively enlarges the pixel size, is highly effective, especially when applied to diffraction data obtained from crystals with small unit cells and measured using multiple-megapixel detectors.

Our research underscores the importance of considering the potential risks associated with particular lossy data-reduction schemes. Specifically, strategies that involve reducing the number of collected patterns or selectively saving determined Bragg peaks may lead to notable deterioration in the quality of the data. Therefore, it is essential to carefully weigh the trade-offs between data reduction and the preservation of crucial scientific information when implementing these schemes.

## 2. Methods and approach

### 2.1. Selection of compression schemes

The topic of data compression is generally a vast and well researched area with many algorithms available for different types of data. Detailed reviews of mostly lossless methods applied to different scientific datasets can be found in the literature (Delaunay *et al.*, 2019 ▸; Duwe *et al.*, 2020 ▸). Here, we focus on data-compression methods applicable to or available in the context of serial crystallography.

Compression schemes typically reduce data volume by exploiting symmetries or redundancies in the data. Thus, the best type of compression to use for a given application depends on the nature of the data being compressed and the information deemed important to retain. Identifying a universal lossy compression scheme is thus difficult since the choice depends on what information must be retained and what can be discarded. For example, high-quality JPEG-type compression is widely used in medical imaging (Wiseman & Fredj, 2001 ▸), and compression factors of 6–8 can be obtained without affecting the diagnosis using conventional JPEG 2000 (Rabbani & Joshi, 2002 ▸; Marone *et al.*, 2020 ▸) and JPEG XR (https://jpeg.org/jpegxr/index.html) compression methods. The appearance of compression artifacts after applying lossy compression is not so important here since JPEG is designed to preserve common visual features of such images. However, JPEG compression is poor for diffraction pattern analysis because the intensity of scientific interest is concentrated in localized Bragg peaks rather than image-like features. In X-ray tomography, azimuthal regrouping of input images followed by CBF byte-offset compression has proven its effectiveness (Kieffer *et al.*, 2018 ▸), thereby exploiting the known rotational properties of a tomographic dataset. However, this assumption often cannot be made for other types of data.

In this paper, we focus our attention on the following compression schemes suitable to reduce the volume of SX data. Specifically, we consider the following.

(1) Lossless compression algorithms commonly available for the HDF5 library including gzip, bzip2, zstd and lz4 with and without bit-shuffle, and different combinations of Blosc (lz4, lz4hc, blosclz, snappy, zlib and zstd).

(2) Data-reduction schemes including non-hit rejection (saving only the diffraction patterns with detectable crystal diffraction), measuring less data (reduced number of frames) and saving only found peaks in diffraction patterns.

(3) Lossy compression schemes including binning (effectively increasing the pixel size and reducing the number of pixels at the detector), quantization (saving only several discrete levels of intensity) and quantization with data rearrangement (saving the intensity value of each pixel re-arranged as a single byte).

We study the effect of each compression type on resultant data and structural model quality obtained with test SFX datasets.

### 2.2. Selection of appropriate data quality metrics

Accurately assessing the potential loss of scientific content resulting from the application of lossy data compression necessitates a comprehensive set of metrics specifically designed to measure the extent of information loss. It is necessary to quantify not only whether the quality of the final molecular structure is affected, but also whether the ability to perform any of the many intermediate analysis steps, for example, peak finding or estimating background signal, is compromised due to loss of data quality.

The determination of macromolecular structures from X-ray diffraction patterns is by now a mature and well developed topic with accepted data quality metrics applied at various stages along the data-processing pipeline (Karplus & Diederichs, 2012 ▸, 2015 ▸; Assmann *et al.*, 2016 ▸). Therefore, we use the following data quality metrics and guides already established for SX and conventional macromolecular crystallography (MX) data processing:

(1) Measured data quality and reproducibility: *I*/σ(*I*), completeness, *R*_split_, CC* or CC_1/2_.

(2) Model refinement quality (*R* values): *R*_free_/*R*_work_.

(3) Visual inspection of the reconstructed electron density maps.

(4) The strength of the anomalous diffraction signal and the ability to perform *de novo* phasing and/or structure refinement.

The first set of metrics evaluates the quality and reproducibility of the merged data and is usually plotted as a function of the resolution (Karplus & Diederichs, 2012 ▸, 2015 ▸). These metrics are also used for selecting an optimal high-resolution cut-off. One important consideration to use these metrics: the resolution of the measured dataset should not be limited by the geometry of the experiment. Although these metrics can offer insights into data quality when structure refinement is unavailable, note that they alone cannot guarantee the successful reconstruction of electron density or the accuracy of any resulting structure (Diederichs & Karplus, 1997 ▸).

The quality of the derived structural maps is of paramount importance for interpreting protein structures. Therefore, it is important to compare how well the derived structure matches the experimental data. For this purpose, the model-refinement *R* value (the second metric from the aforementioned list) measures the agreement between the observed and calculated structure factor moduli (Brünger, 1992 ▸; Karplus & Diederichs, 2012 ▸). Going one step further, protein crystallographers introduced the *R*_free_ metric which measures how well a subset of data omitted from the refinement process is explained by the final model to indicate possible over-fitting or incorrect results.

Ultimately, the quality of the reconstructed map is interpreted by an expert. Here, it is occasionally observed that refinement quality assessed by *R* values alone can lead to incorrect conclusions about the model quality. For example, expert inspection of the structure may reveal nonphysical chemistry such as overlaps of atoms or bonds, or conversely the presence or absence of extra electron density such as structural solvent which must be included in the structural model to further improve agreement with the experiment. Thus, the third method of visually inspecting the quality of the reconstructed electron density is vital. Unfortunately, it is not so easy to automate this method, and it requires an expert to decide if the electron density looks reasonable.

Therefore, we turn to an additional method based on the strength of the anomalous signal and the ability to perform *ab initio* reconstruction of the structure as an effective method for determining whether information has been lost during data manipulation. The anomalous signal in single/multi-wavelength anomalous diffraction (SAD/MAD) datasets is usually weak, and the method of *de novo* phasing can work only if the error in the determination of the structure factors is lower than the Bijvoet differences. This method of structure determination is known to require a higher data quality than, for example, molecular replacement, making it a good test of whether lossy compression has adversely affected the data. To further increase the sensitivity of this approach, we start with a subset of the data that is only large enough for the *de novo* phasing pipeline to work, so that any meaningful deterioration in data quality will lead to an inability to reconstruct the structure (Nass *et al.*, 2020 ▸).

We have used all four quality checks to test different lossy compression algorithms. Some applications, such as binding screening studies, may be more robust to small data errors and suffice if *R*_free_/*R*_work_ are exceeded (metric 2), whereas cutting-edge cases such as *de novo* structure determination of sensitive time-resolved structural studies may require a higher data fidelity such as those measured by anomalous differences (metric 4). We further hope this suggested set of metrics and guides will be used to compare other compression schemes not studied here, ideally using the same open datasets.

### 2.3. Selection of test datasets

An ideal protein crystal diffraction pattern measured using a noiseless detector would be very sparse, consisting of bright Bragg peaks with zero background. Such a diffraction pattern is easily compressible by most existing lossless compression algorithms. By contrast, in real crystallography experiments the background recorded in each diffraction pattern is quite high and is often comparable to the strength of the measured Bragg peaks. Statistical noise in the background leads to significant intensity differences between neighboring pixels. Furthermore, the integrating detectors used at XFELs do not count incoming photons but rather accumulate the charge deposited in a single femtosecond-duration exposure. Accumulated charge including intrinsic electronic noise sources is not directly converted to individual photon counts but estimated after the detector is read out. Experience shows that compression of experimental SX diffraction data rarely reaches a compression factor better than 5 using lossless algorithms.

To capture these challenges, we selected four representative SX datasets covering a range of detector technologies (both counting and integrating), photon sources (including synchrotron and FELs) and varied sample-delivery methods. These methods include tape drive (Henkel *et al.*, 2023 ▸; Zielinski *et al.*, 2022 ▸), lipidic cubic phase (LCP) jet (Weierstall *et al.*, 2014 ▸) and gas dynamic virtual nozzle (GVDN) jet (DePonte *et al.*, 2008 ▸). The dataset specifics are enumerated in Table 1 ▸. Notably, three of the datasets listed are available for download from the CXIDB (Maia, 2012 ▸), and the associated code, deposited in GitHub (https://github.com/galchenm/binningANDcompression.git), facilitates the examination of all compression methods detailed in this study. The selection of tested samples (Table 1 ▸) is purposeful, aiming to evaluate diverse algorithms across different unit cells. Detailed unit-cell parameters and space groups can be found in Table S1 of the supporting information.

These test cases cover a representative sample of current protein crystallography datasets, including cryo-crystallography, where the background is high and a counting detector is usually used (similar to the second test dataset). In our discussion, we have selected comparisons between datasets that we found to best illustrate the challenges posted for different algorithms based on our practical experience working with different compression schemes and datasets. This avoids the combinatorial explosion of testing all algorithms against all datasets, enabling us to focus our attention on the key issues rather than presenting large tables of exhaustive comparisons.

## 3. Results

### 3.1. Lossless compression

Lossless compression approaches are commonly used for compressing scientific data. By definition, no information is lost and the original data can be restored verbatim. The only question is therefore the achievable compression rate (CR) and speed, which are both highly dependent on the statistical properties of the data to be compressed.

We observe that lossless compression schemes vary significantly in effectiveness depending on the experimental SX data to which they are applied. From our sample datasets, two extreme cases for lossless compression were the EIGER 16M detector and the AGIPD. The EIGER 16M is a counting detector that registers zero counts without incident photons and integer values corresponding to the number of photons incident on a pixel. On the other hand, the AGIPD is an integrating detector with three different gain stages for which calibrated data are stored in a floating-point format, and the value in a pixel might be even negative due to the subtraction of non-constant dark signal. For integer data (EIGER 16M, CS-PAD or AGIPD rounded to integer values), compression ratios of higher than 4 can be achieved using zstd or bzip2, and in some cases can be higher than 10. Commonly available in HDF5, gzip compression (level 6) demonstrates quite good results. Conversely, the achievable compression ratio for floating point type data (AGIPD detector) reaches only 1.3 with gzip level 6.

A complete table with the results of the lossless compression algorithms tested against our reference datasets can be found in Table S2. We observe that the conversion to photons (integers) is an important factor in determining compression efficiency even if this conversion is itself a form of lossy compression, as expected.

Another important parameter for lossless compression is speed, which is especially critical for online data processing and real time compression (Fig. S1 of the supporting information). The tests were performed using blocks of 1000 frames from the two extreme cases above: EIGER 16M and AGIPD 1M detectors. The results indicate that most of the tested compression algorithms demonstrated similar performances, with the exception of bzip2, which usually produces the best compression ratio. But the best compromise of the compression/decompression speed versus compression ratio was observed for the blosc, zstd and bit-shuffle algorithms. For more information, see Fig. S1.

### 3.2. Rejection of non-hits (vetoing)

In serial crystallography, it is common that not all detector frames contain crystal diffraction. This is due to the sample being passed across the X-ray beam and intersecting with the beam at random. Whether any individual detector frame contains diffraction is a matter of chance. The hit rate, namely the fraction of measured frames containing useful crystal diffraction, is one of the important characteristics of an SX experiment and is related to the sample concentration, flow rate and relative size of the X-ray beam, among other factors. In practice, the hit rate during experiments is frequently rather low, typically ranging between 0.1 and 10%. This observation leads to the obvious conclusion that the volume of data can be reduced by discarding data frames without any crystal diffraction.

The urgent question is how to save collected data: do we need to store only frames containing obvious crystal diffraction, or should all the data be retained? Could we expect that more advanced data-processing algorithms could help to obtain better results in the future? To answer these questions, we re-processed the first high-resolution SFX dataset measured in 2011 (Boutet *et al.*, 2012 ▸) using a recent SX processing pipeline (*CrystFEL* version 0.10.1 and *Phenix* version 1.13). This reprocessing was performed in three different ways.

(1) Using the data as deposited at the CXIDB for the original paper (third row in Table 1 ▸).

(2) Repeating processing from raw data archived to tape for all measured data using modern detector calibration and hit-finding methods applied to all data frames and re-processed with the new algorithms.

(3) Repeating processing as described in (2), but using only the same data frames as deposited for the original paper.

The structure refinement of reprocessed data were performed with *phenix.refine* (*Phenix* version 1.13) with parameters such as ‘xray_data.high_resolution = 1.6’ and ‘xray_data.low_resolution = 20’ using PDB entry 6ftr (Wiedorn *et al.*, 2018 ▸) as the search model. The results are summarized in Table 2 ▸.

**Figure 1:**
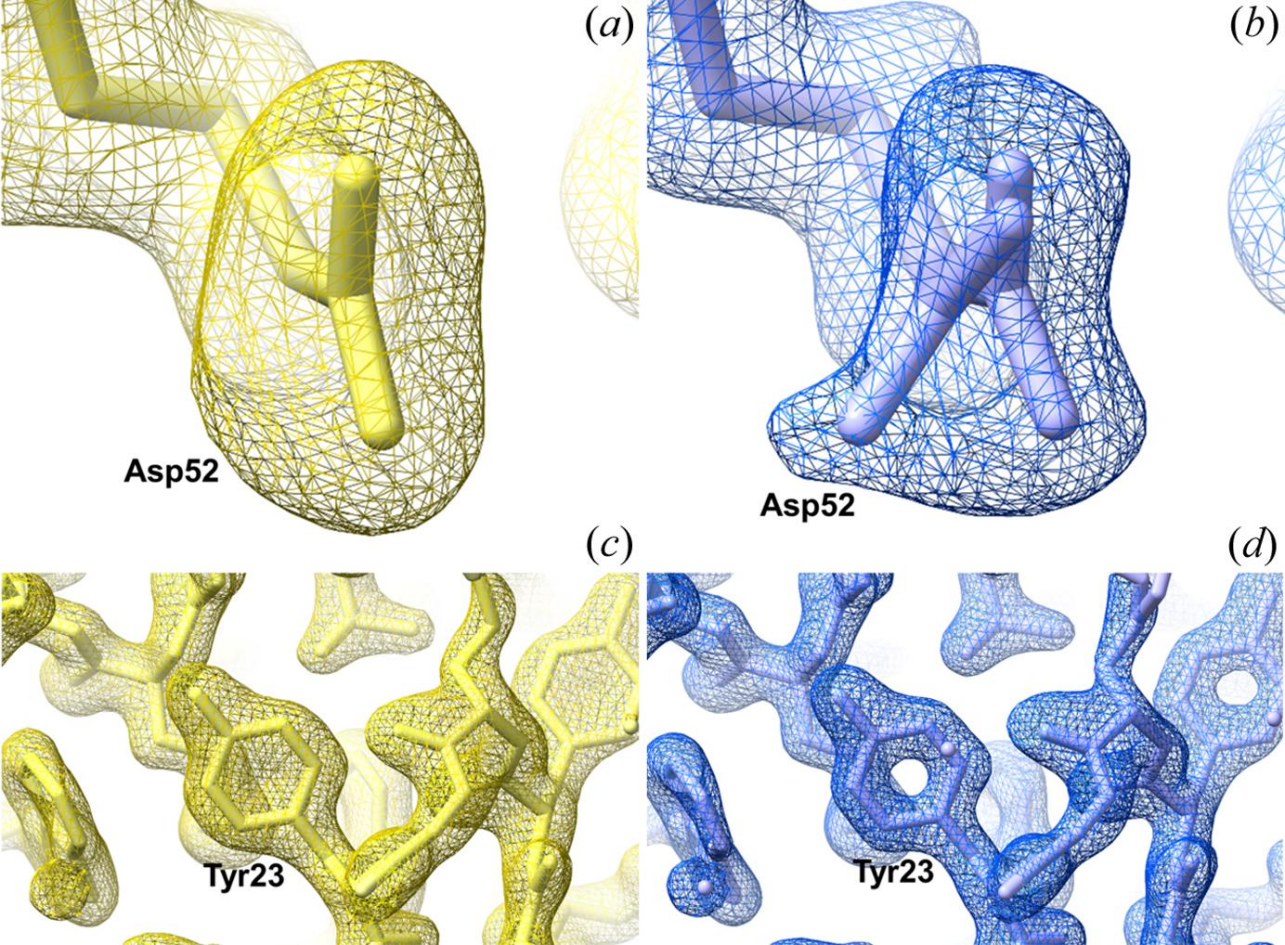
Comparison of electron density maps (2*F*_o_ − *F*_c_, contour level σ = 1.5) of lysozyme in the original structure (PDB entry 4et8; 1.90 Å yellow mesh and model) and the reprocessed data using all frames (1.49 Å, blue mesh and model). (*a*) and (*b*) Active-site residue Asp52 could be modeled with an alternative conformation using the reprocessed data. (*c*) and (*d*) Another section of the structure around Tyr23 with the same maps as described above (but with contour level σ = 0.8) shows more detailed density for the aromatic amino acids when using the reprocessed data.

From Table 2 ▸ we can see that the original paper reported lower-quality results than after any re-processing of the data performed now. Note that the results in Table 2 ▸ are limited by the geometry of the experiment – the resolution of 1.49 Å corresponds to the corners of the detector (Fig. S2). Determining the resolution cut-off point is primarily a subjective process. However, we consider all the quality metrics mentioned above in order to reach a reasonable compromise. The reconstructed electron density and the structural model between the original and the re-processed data are shown in Fig. 1 ▸ (additional examples can be found in Fig. S3). One example given (Asp52) is an active-site residue essential for the enzyme mechanism of lysozyme (Held & Van Smaalen, 2014 ▸). Re-processing data results in electron density maps that allow us to resolve alternative conformations of Asp52, which allows more accurate interpretation of biological function. The quality improvement is not surprising since Boutet *et al.* (2012 ▸) reported the first-ever high-resolution SFX structure, and data-processing software has significantly improved in the following years. There are several reasons for the improvement in result quality: new algorithms for indexing (including indexing multiple crystals per pattern) and integration implemented in *CrystFEL* (White *et al.*, 2016 ▸), better knowledge of the detector geometry (Yefanov *et al.*, 2015 ▸) and a different strategy for background subtraction (see Fig. S4). Note that the re-processing of the deposited MTZ file (PDB entry 4et8) using a more recent version of *Phenix* did not result in substantial improvements in the reconstructed structure: published *R*_free_/*R*_work_ = 0.229/0.196 versus *Phenix* (version 1.20) *R*_free_/*R*_work_ = 0.2109/0.1730 (additional comparison of the results achieved using different versions of *Phenix* are presented in Table S3). Based on this observation, we can conclude that the significant improvement observed in the reconstructed structure can be attributed to the processing of actual diffraction frames.

An important observation is that an improved identification of frames containing crystal diffraction (the ‘hit finding’ step) did not lead to a significant improvement in data quality. This is because hits are identified based on the simple metric of the presence of Bragg peaks. Most of the additional patterns found by repeating hit finding on all raw data only served to identify frames containing weak diffraction corresponding to small crystals or crystals hit by the tail of the X-ray beam. Weak patterns provide less information than the strong patterns found in the initial analysis, especially at high resolution, thus contributing little to improving the quality of the refined structure.

As a check, we reprocessed several other datasets from the same beam time as well as from some other LCLS beam times (not reported in detail here). Comparison with the originally published datasets always demonstrated that new processing would lead to significantly better results: we have observed the improvement of the achievable resolution by up to 1 Å: from 3.5 to 2.5 Å in the case of a protein with a large unit cell (article in preparation). However, repeating the selection of data frames to process (hit finding) usually resulted in marginal improvement, if any.

We therefore conclude that for strongly diffracting crystals it is indeed a good compromise to save only frames with clear diffraction peaks but to save these data in the ‘raw’ data format so that improvements in detector calibration can be applied from the raw data later.

For the case of weakly diffracting crystals, the situation is more complicated because there is always the potential that weakly diffracting frames may not be found (Ayyer *et al.*, 2015 ▸) and additional information may be identified in the diffraction patterns after the experiment, such as diffuse scattering outside of Bragg peaks (Ayyer *et al.*, 2016 ▸). The decision as to whether this justifies the retention of all weakly diffracting data is one that each experiment team will have to make.

### 3.3. Measuring less data

Measuring more data frames in an SX experiment generally leads to higher-quality results due to averaging a greater number of observations. But an associated question during any experiment is: when have sufficient data been collected to answer the scientific question at hand? Measuring only enough data to answer a scientific question reduces experiment time and minimizes the amount of data collected to only the amount needed. Indeed, one of the common questions during SX beam time is when to stop data collection. In order to address this question, it is necessary to assess the impact of reducing the number of measured frames (Tolstikova *et al.*, 2019 ▸; Zielinski *et al.*, 2022 ▸).

**Figure 2:**
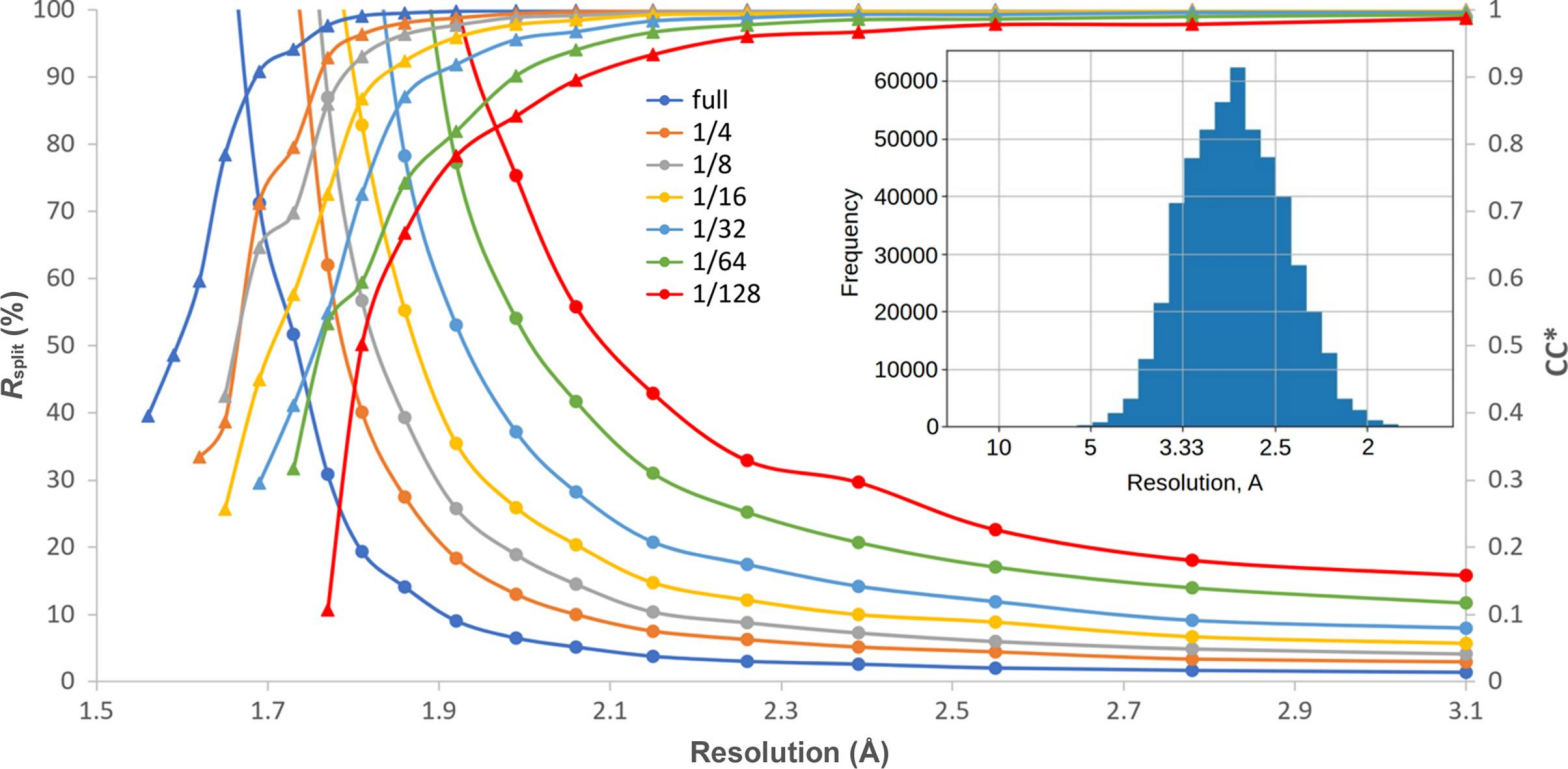
Data quality metrics CC* (from 0 to 1, higher is better) and *R*_split_ (up to 100%, lower is better) for the different fractions of the measured dataset of lactamase (first row in Table 1 ▸). The insets show the histogram of achievable resolution for each pattern.

The effect of measuring fewer data frames can readily be checked for any single dataset by integrating progressively smaller data subsets. Fig. 2 ▸ shows the quality metrics CC* and *R*_split_ versus resolution as well as *R*_free_/*R*_work_ metrics for the different subsets of lactamase data measured during one of the tape-drive (Beyerlein *et al.*, 2017 ▸; Henkel *et al.*, 2023 ▸; Zielinski *et al.*, 2022 ▸) SX experiments at the P11 beamline of PETRA3. The initial dataset consists of 200 000 diffraction patterns and is processed as smaller subsets equivalent to less measurement time (Table 3 ▸). This dataset was collected as a single 25 min acquisition with an EIGER2 X 16M detector operated at 133 Hz.

The degradation in data quality with fewer patterns is rather obvious from Fig. 2 ▸ and is to be expected given the fact that redundant measurements improve statistical metrics such as CC*/*R*_split_ and thus the quality of the obtained data. We can conclude that while the strategy of limiting measurement time is understandable, measuring more data always improves data quality in line with known statistics. Therefore, the lossy reduction idea to save space just by measuring fewer data is, in fact, not optimal because it results in lower quality (see Fig. 2 ▸). On the other hand, the improvement of the resolution achievable using 1563 patterns (1/128) versus all 200 000 patterns is from 1.8 to 1.58 Å (0.22 Å difference). Therefore, the decision to halt data acquisition should be made based on the specific scientific inquiries of the study.

For the evaluation of data quality corresponding to the number of indexed lattices, the stream after ambigator (White *et al.*, 2016 ▸) was split randomly at 1/4, 1/8, 1/16, 1/32, 1/64 and 1/128 and then subjected to scaling and merging. *Phenix* (Adams *et al.*, 2010 ▸) (‘phenix.reflection_file_editor’) was used to add the same set of *R*_free_-flags to each resulting dataset, and all datasets were refined with *phenix.refine*, using the same starting model, parameters and resolution cut-off (as set by the highest-resolution shell still containing useful data for the 1/128 dataset). *Polygon* (Urzhumtseva *et al.*, 2009 ▸), *MolProbity* (Davis *et al.*, 2007 ▸) and *Coot* were used for valid­ation of the final model.

### 3.4. Storing only detectable Bragg peaks

Another proposed data-reduction scheme is to save only the information around peaks found in each measured diffraction pattern. The idea is that only Bragg peak information affects the structure, so it should only be necessary to save information around the Bragg peaks.

Fig. 2 ▸ shows that adopting such a strategy will limit the achievable resolution. For this dataset, if we limit ourselves to only using the found peaks, the achievable per pattern resolution would reach 2 Å (5 nm^−1^) for only a very small number of patterns (see the resolution histogram in the inset), while the entire dataset achieves a resolution of 1.58 Å according to CC* cut-off decision. It is well known that redundant measurement of weak data improves the overall resolution achieved beyond the resolution, at which peaks can be detected before integrating (Gati *et al.*, 2017 ▸). As a consequence, compression schemes based on saving full detector data only around detectable peaks (Underwood *et al.*, 2023 ▸) will artificially limit the resolution. For example, by processing the stream file from Table 2 ▸ to include only reflections from each pattern found in the initial peak search, the resulting resolution dropped to 1.62 Å and *R*_work_/*R*_free_ increased to 0.236/0.292. In other words, we conclude that retaining data from only detected peaks noticeably decreases the structure quality.

### 3.5. Binning to lower the number of detector pixels

Reducing the pixel count by binning data to fewer pixels is commonly used when it is known that the detector has a finer pixel pitch than is strictly necessary for the current measurements. For example, when the beamline is equipped with a 16M detector but a 4M detector would suffice for the experiment, it is possible to bin the data after measurement. Alternatively, if the detector is capable of selectively recording a specific region of interest (ROI), and the beamline geometry permits moving the detector closer to the sample, it becomes feasible to save solely the designated small ROI. In both cases the separation between Bragg peaks has to remain adequate for data processing and the shape of the Bragg peaks need not be resolved. For experiments with a monochromatic X-ray beam, such minimum distance should be on the order of 5–10 pixels. Therefore, many datasets, especially for proteins with small unit-cell parameters, can be binned.

Binned data (Table S4 and Fig. S5) indicate that 2 × 2 pixel binning for the tested datasets measured with 16M detector did not degrade the data quality for the samples we used, but the data volume was reduced by a factor of 3–4. The one caveat is that it might be more difficult to detect the peaks after binning; therefore, we have developed a procedure in which the positions of the peaks are found before binning, recalculated into the coordinates of the binned image and saved within the output HDF5 file. Those saved positions can be later used for indexing.

### 3.6. Quantization of detector output

The quantization of data refers to the reduction of the bit depth of the saved data to a lower number of discrete values, reducing unnecessary precision in the stored values such as remapping 32 bit integers to 16 or 8 bit values, or converting floating point data to integers. In photon science, a common form of quantization is the conversion of the electrical signal to photon counts. This is performed in the electronics of counting detectors (PILATUS, EIGER), where each pixel directly counts the number of incident photons at high speed. As previously noted, such data compresses well using lossless compression schemes compared with data saved in floating point format. And, in general, data with fewer discrete values compress better using most of the lossless compression schemes.

Since counting detectors are not suited for the short pulse lengths found at XFEL sources, integrating detectors that integrate the deposited charge in each pixel during the exposure are used. Converting the deposited charge into the number of incident photons helps to reduce the data precision required; however, this operation relies on good calibration of detectors and is not necessarily a trivial task, there is a tendency to save actual digitizer readout for later photon conversion.

Our tests on quantization indicate that reducing the data precision of integrating detectors can be highly effective at enabling data compression. Results are presented in Table S5, where we test not only conversion to photons but also more aggressive reduction of data precision. For the AGIPD detector, even a quite high quantization level [1024 analog-to-digital units (ADUs) per quantum, which corresponds to approximately 14 photons at 9 keV] still achieves reasonably good data quality: *R*_free_/*R*_work_ of 0.1753/0.1543 with a compression ratio of 64, compared with *R*_free_/*R*_work_ of 0.1670/0.1497 for the original data.

To assess the impact of quantization in comparison with the previously outlined strategy of collecting a reduced dataset, we conducted the following test: quantization to 64 and 1024 ADUs was applied for both the entire dataset (comprising 190 000 diffraction patterns) and its 1/16 fraction (refer to Table S6 and Fig. S6). Although the volume of the 1/16 subset was nearly equivalent to the volume of the data rounded to 1024 ADUs, the data quality was superior in the latter dataset. This is evident in the higher achievable resolution and improved statistics even at lower resolutions (see Fig. S6). The findings demonstrate that prioritizing the acquisition of a sufficient number of patterns is more important than precise recording of the diffracted intensities.

### 3.7. Non-uniform quantization

An even higher compression ratio can be achieved by selecting the levels for quantization in a non-uniform way. Diffraction from crystals usually consists of some background (typically smooth) and rather sharp Bragg peaks. As mentioned earlier, a high dynamic range is usually needed to record such diffraction, with the intensity of the Bragg peaks varying from rather high (at low resolution) to very low (at high resolution). However, though single photon counting may be useful in weak reflections, it is not necessarily needed in the bright Bragg peaks. For this reason, special X-ray detectors (AGIPD, JUNGFRAU, ePIX) were developed that have variable gains per pixel to be able to record single photons at low flux as well as very high intensities (up to 10 000 photons per pixel) at high flux in a single image.

**Figure 3:**
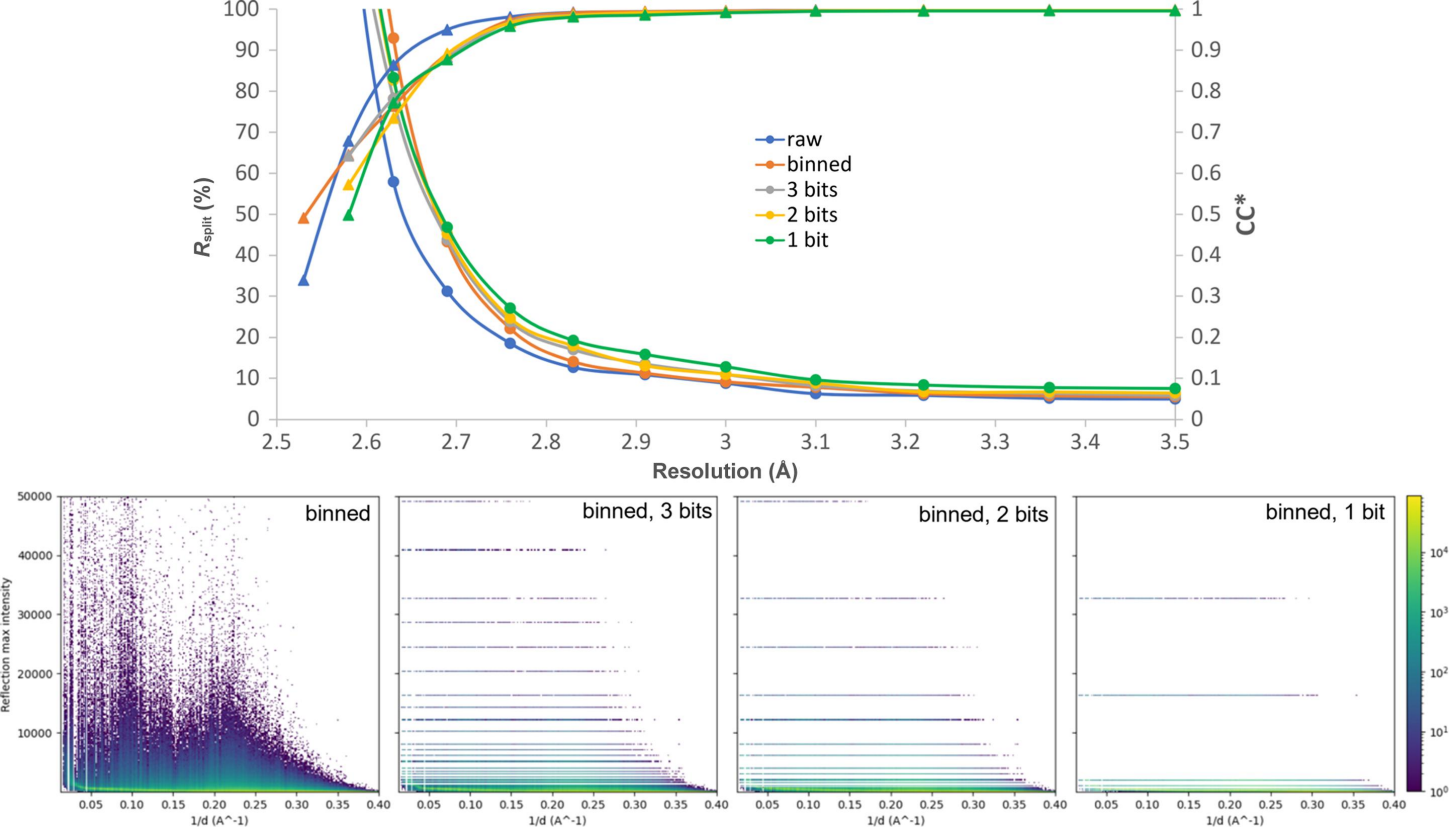
Plot of quality metrics *R*_split_ and CC* for the original data of thaumatin (see Table 1 ▸), binned and rounded to 1, 2, 3 of the most significant bits. Under the plot, the histograms of found peak intensities over 1/*d* (peakograms) for different datasets are presented.

The tolerable relative error of the peak intensity drives the required precision. Thus, at low photon counts the quantization levels are rather dense, while at the higher fluxes, the levels are comparatively sparse, in proportion to the counting noise. In the measured data this can be achieved by keeping the value of just a few of the most significant bits (starting from the first non-zero bit) in the integer representation of the measured intensity. For positive values, the simplest method is to preserve the most significant bit with the value of ‘1’ and set all other bits to 0. To obtain better results, rounding to the nearest value of 1, 2 or 3 most significant bits is utilized instead of truncation (see the examples in Table S7). The histograms in Fig. 3 ▸ provide an illustrative example of pixel intensities at different distances from the center of the detector. Note that this rounding technique alone does not decrease the data size. However, the modified data become highly compressible using various lossless compression algorithms (as indicated in Table 4 ▸).

The proposed compression applied to the data discussed previously had almost no influence on data quality metrics such as CC* or *R*_free_/*R*_work_ (see Table S8). Therefore, for this test, we have chosen the technique that is much more sensitive to data quality: SAD. We have used the thaumatin dataset (second row in Table 1 ▸). The structures for different datasets after applying lossy compression algorithms of thaumatin were solved by SAD phasing and refinement using the *Crank2* pipeline (Skubák & Pannu, 2013 ▸) with default settings. As can be seen from Fig. 3 ▸, the quality of the data did not degrade much after applying rounding to several of the most significant bits. The results presented in Table 4 ▸ demonstrate that even the SAD data can still be used successfully if only two significant bits are saved (more statistics can be found in Table S9 and the reconstructed structures in Fig. S7). At the same time, saving just the single most significant bit is not enough for the same dataset – as can be seen from the last row of Table 4 ▸, the *R*_free_/*R*_work_ are very high in this case.

Retaining only the most significant bits is quite similar to the way the data are represented by floating-point numbers, thus we have also represented the integer data in a floating-point-like way – we have converted the 32 bit integers into 8 bit floating-point values: one bit for the sign, 5 bits for the exponent and 2 more bits for the mantissa. From the numbers in Table 4 ▸, one can see that such conversion allows one to compress data even better. One very important benefit of the proposed lossy compression scheme is its speed. The truncation of the least significant bits requires very little computation. Indeed, the conversion may lend itself to implementation directly in hardware, such that it could even be realized within the detector.

## 4. Discussion

Several of the data-reduction techniques described above are already in use by various research groups, indicating some level of acceptance of the compromises involved. For example, users typically copy only diffraction patterns that contain diffraction (hits) after the SFX experiments at LCLS, SACLA or the European XFEL. This is done in the knowledge that the facility stores the entire raw data for 10 years. However, even at synchrotron facilities such as APS or ESRF, raw data are ultimately deleted after some time. We often collect data using the JUNGFRAU 1M detector at 1 kHz speed, resulting in up to 50 TB of raw files per experiment. After hit-finding and lossless compression, we copy only 2–5 TB of data and delete everything else. This strategy does not adversely affect data quality, as justified by our tests.

For multi-megapixel detectors such as the EIGER 16M or JUNGFRAU 16M, binning data to a smaller detector is effective provided the crystal unit cell permits sufficient separation between Bragg peaks. We use this approach together with saving only hits for our SSX experiments performed at the P11 beamline of the Petra III synchrotron, where we routinely achieve a compression ratio of 5–7 from binning and non-hits rejection on top of the compression factor of 5–6 achieved by the bit-shuffle filtered LZ4 compression used by Dectris or gzip. This gives a total compression ratio of up to 40 times compared with saving raw data, with no noticeable degradation in scientific output. Currently, the optimization of hit-finding parameters requires some human interaction, but it should be possible to eventually automate this step.

Even higher CRs are possible by quantizing the detector output into fewer discrete levels. This method works well for SX data because SX relies on statistical measurements: in effect, it is more important to measure more patterns than to measure the intensity in each pattern more precisely. This is because the error in the determination of the exact integrated intensities of the Bragg peaks is rather high for SX, mostly due to the unknown partiality and possible error in the determination of the precise crystal orientation (Kirian *et al.*, 2010 ▸; White, 2014 ▸; Spence, 2020 ▸). Additionally, one can perform the quantization in a non-linear way to have finer increments at low intensity and coarser increments for strong signals following noise on counting statistics. In this way, the low signal data are saved almost without losing the precision which is very important for data measured at high resolution close to the detector edges.

Conversely, saving data only around found Bragg peaks results in a significant loss of data quality. The final model resolution often extends beyond found peaks due to the presence of a weak signal at Bragg peak locations in individual diffraction patterns which nevertheless integrates above noise levels when many observations of the same reflection are averaged. Alternatively, saving only predicted peak positions necessitates flawless indexing – a requirement that, as illustrated in this paper, is not consistently met. Moreover, relying solely on the preservation of predicted peaks fails to leverage the advantages offered by emerging indexing algorithms. Similarly, reducing the measurement time and, thus, the number of measured diffraction patterns reduces data following statistics: fewer measured patterns means fewer observations of each reflection and thus a reduction in signal-to-noise of the merged reflection intensity.

So far, this discussion has considered only methods to reduce the volume of raw data frames by compressing or rejecting individual diffraction patterns. Even better data reduction can be achieved if the original diffraction patterns are discarded and only intermediate calculation results are retained. For example, in rotational crystallography it is common to look at only the resulting merged reflection data and the original diffraction patterns are almost never revisited. Similarly, efforts are underway in SX to perform all indexing and integration in real time, obviating the need to ever save individual diffraction patterns. If this can be done, the compression ratios achieved can be enormous – instead of many terabytes of raw files, less than 10 MB are saved. This approach is usually applied during or after the experiment (minutes to months delay). In this case, however, it is not possible to revisit the original data at a later time. Such approaches can only be adopted when there exists a very well established pipeline, and all calibration factors, including the geometry of the experiment (Yefanov *et al.*, 2015 ▸) and the detector response, are very well known. Although this is not yet the case for SX experiments, an investment in robust geometry and detector calibration combined with an established analysis pipeline could significantly reduce saved data volumes in the future. It could be applied if the costs for eventually re-doing the experiment are lower than those for the storage of data.

However, we have also shown that software improves over time and careful reprocessing of previously collected data might deliver much better results at a later point in time. For example, we showed that reprocessing the lysozyme dataset measured in 2011 leads to a much higher resolution than originally obtained, provided the raw frames containing crystal diffraction were available. Even some structural features, that were not observed during the initial analysis, were resolved after the reprocessing. The question of how much data should be stored and for how long is undoubtedly a matter of debate that will continue for quite some time.

Finally, we note that the compression schemes described in this paper should also be applicable to protein crystallography with electron or neutron diffraction. Some of the methods can be useful for other techniques that use diffraction and 2D detectors. However, as each analysis chain is different, it is hard to generalize and the effect of compression or data reduction on each technique should be considered separately.

## 5. Conclusions

Although it is currently still technically feasible to save all detector output, the cost of doing so is continually growing as X-ray detectors become faster and the number of pixels is increasing. At some point, it will therefore be necessary to decide what data to retain. Our analysis here in the context of SX shows that lossless compression alone is of some use in reducing data volumes, but is highly dependent on the detector and experiment and is ultimately limited in the CRs that can be achieved.

To assess the effect of lossy compression schemes, we used a set of data metrics capable of assessing the loss of information due to the application of various compression schemes. This required a careful understanding of the specific analysis techniques but is nevertheless an essential step in evaluating different compression algorithms. We checked metrics such as data quality and reproducibility (signal-to-noise ratio, *R*_split_, CC*), the quality of the reconstructed structure factors (*R*_free_/*R*_work_), and the possibility to use the anomalous signal for *ab initio* structure reconstruction (SAD phasing). It is of utmost importance to employ each of the data quality metrics mentioned in a proper manner. For example, one has to ensure that the achievable resolution is not limited by the geometry of the experiment (detector edge resolution). Failure to address such limitations may render certain quality metrics insensitive to potential degradation in data quality.

We find that saving the raw detector frames containing strong crystal diffraction is highly effective when it comes to reproducing results at a later stage. Discarding blank frames has little effect on data quality even if some of those ‘blank’ frames may be found to contain weak diffraction using more advanced algorithms developed at a later time. Conversely, retaining information from only locations of found Bragg peaks in each pattern has a significantly detrimental effect on data quality.

Lowering the number of pixels in the detector is an obvious saving of data space provided it is compatible with the crystal being studied. Where data are saved in floating point ADUs, quantization to integer numbers of photons is highly effective in reducing data volumes as the additional precision of sub-single-photon counting accuracy is not required for SX measurements. Also, compression of the dynamic range of measurements in a non-linear manner following statistical noise is highly effective, so that weak reflections are still accurately measured, but there is less precision in the measurement of strong intensities. This is achieved in practice by saving only several of the most significant bits of the values measured by each pixel. In this way, the low signal data are saved almost without losing the precision, which is very important for data measured at high resolution close to the detector edges. In principle, such a scheme is similar to the multiple-gain mode used in modern detectors for capturing high dynamic range signals while keeping high sensitivity for low signals but applied with many more levels in the software after the measurement is made. This data-reduction approach is very computationally efficient, therefore it might be implemented inside new detectors.

By combining the above data-reduction methods including real time hit finding, binning, quantization to photons and non-linear reduction of the dynamical range, it should be possible to continue retaining individual detector frames for later study while also reducing the volume of data which must be permanently retained at facilities or user groups worldwide.

## Supplementary Material

Supporting tables and figures. DOI: 10.1107/S205225252400054X/if5003sup1.pdf

## Figures and Tables

**Table 1:** Datasets used for the different tests

Facility (beamline)	Energy of X-rays (keV)	Detector	Data type	Sample delivery	Background level	Samples	PDB entry, CXIDB ID	Reference
Petra III (P11)	12	EIGER2 16M (counting, photons)	Integer	Tape drive	Low	Lysozyme, lactamase, ferritin, MPro	–	Unpublished
SwissFEL (Alvra)	4.57	JUNGFRAU 16M (integrating, photons)	Integers	LCP	High	Thaumatin	6s19, CXIDB 104 (runs 12–41)	Nass *et al.* (2020 ▸)
LCLS (CXI)	9.4	CS-PAD 2.3M (integrating, ADUs)	Integers	GDVN	Moderate	Lysozyme	4et8, CXIDB 17 (runs 305–396)	Boutet *et al.* (2012 ▸)
EuXFEL (SPB)	9.3	AGIPD 1M (integrating, ADUs)	Floating point	GDVN	Moderate	Lysozyme, granulovirus	6ftr, CXIDB 98 (runs 96–98)	Yefanov *et al.* (2019 ▸)

**Table 2:** Different processing of the lysozyme dataset from 2011

Dataset	Hits/indexed /crystals	Resolution limit (Å)	*R*_split_/ Completeness	*R*_free_/*R*_work_
Originally published results (CXIDB ID 17)	66442/12247/12247	1.9	0.158 (NA)/98.3% (96.6%)	0.229/0.196
Fully reprocessed all frames from raw data	108814/71488/137074	1.49	0.029 (0.15)/100% (100%)	0.189/0.168
Fully reprocessed same frames as original	66442/59070/123977	1.51	0.029 (0.14)/99.9% (97.87%)	0.195/0.172

**Table 3:** The impact of the number of measured diffraction patterns on the final data quality Different fractions of the original lactamase dataset (200 000 patterns) were processed.

Part	No. of patterns/hits	Indexed patterns/crystals	*R*_work_/*R*_free_
All	199606/198088	187826/505329	0.1561/0.1881
1/4	49902/49531	46947/126301	0.1576/0.1866
1/8	24951/24759	23477/63191	0.1603/0.1936
1/16	12475/12387	11759/31731	0.1688/0.1944
1/32	6238/6193	5888/15859	0.1728/0.2048
1/64	3119/3098	2929/7895	0.1794/0.2122
1/128	1559/1550	1450/3968	0.1932/0.2202

**Table 4:** Lossy compression of SAD data of thaumatin CR achieved using gzip (compression level 6) and shuffle.

Type	CC_ano_	*R*_split_/CC*	*R*_work_/*R*_free_	No. of residues	CR	CR, 8 bits
Raw	0.327	5.97%/0.998	0.223/0.282	209	1	–
Binned	0.320	6.35%/0.998	0.217/0.283	208	5	–
Binned, 3 bits	0.247	6.65%/0.998	0.238/0.299	205	32.18	36.34
Binned, 2 bits	0.271	6.81%/0.998	0.270/0.334	205	38.84	45.06
Binned, 1 bit	0.251	7.94%/0.998	0.531/0.551	181	49	59.28
