# Supplementary material for: Data reduction in protein serial crystallography
Source: IUCrJ. 2024 Feb 8;11(Pt 2):190–201. doi: 10.1107/S205225252400054X (PMC10916297; doi:10.1107/S205225252400054X)
Supplement: Supplementary file 1 [file m-11-00190-sup1.pdf]

IUCrJ

Volume 11 (2024)

Supporting information for article:

Data reduction in protein serial crystallography

Marina Galchenkova, Alexandra Tolstikova, Bjarne Klopprogge, Janina Sprenger, Dominik Oberthuer, Wolfgang Brehm, Thomas A. White, Anton Barty, Henry N. Chapman and Oleksandr Yefanov

Table S1 Information about samples used for different test

**Sample**	**Unit cell parameters**	**Space group**
Lysozyme (lyso)	79.2 79.2 38 90 90 90	P 43 21 2
Lactamase (lacta)	41.8 41.8 233.3 90 90 120	P 32 2 1
Ferritin	181 181 181 90 90 90	P 2 3
Granulovirus polyhedrin (gv)	103.4 103.4 103.4 90 90 90	I 2 3
SARS-CoV-2 Main protease (Mpro)	114.7 53.8 45.1 90 101.86 90	C 1 2 1
Thaumatin (thau)	58.5 58.5 151.3 90 90 90	P 41 21 2

Table S2 The evaluation of available lossless compressions, available through h5py (gzip, lzf), PyTable (bzip2), and h5plugin (Blosc+{lz4, lz4hc, blosclz, snappy, zlib, zstd}; bitshuffle with/without lz4; lz4; zstd), on different datasets. The compression rate (CR) is presented for the original data as well as for the same data after applying some of the lossy compressions. 1bit and 3bits – refer to the lossy compression with leaving only 1 or 3 the most significant bits.

	**AGIPD**			**Eiger 16M**		
	**float, 32bit**	**int, I16**	**int, I32; 3 bits, min=512**	**int, U16**	**int, I32; 1 bit, min=1**	**int, I32; 3 bits, min=1**
**Bitshuffle with lz4**	1.103	3.345	8.25	3.581	9.939	7.805
**Blosc,blosclz,level = 6, bitshuffle**	1.118	2.837	6.361	3.571	10.476	7.897
**Blosc,blosclz,level = 6, shuffle**	1.095	1.88	3.453	2.194	8.388	5.325
**Blosc,blosclz,level = 6,w/o shuffle**	1	2.335	2.091	1.986	3.781	2.48
**Blosc,blosclz,level = 9, bitshuffle**	1.118	2.837	6.361	3.571	10.476	7.897
**Blosc,blosclz,level = 9, shuffle**	1.095	1.927	3.453	2.267	8.388	5.325
**Blosc,blosclz,level = 9,w/o shuffle**	1	2.335	2.091	1.986	3.781	2.48
**Blosc,lz4hc,level = 6, bitshuffle**	1.138	3.038	7.295	3.784	11.502	8.29
**Blosc,lz4hc,level = 6, shuffle**	1.156	2.379	5.181	2.671	11.453	6.464
**Blosc,lz4hc,level = 6,w/o shuffle**	1	3.06	5.298	2.275	7.235	4.147
**Blosc,lz4hc,level = 9, bitshuffle**	1.139	3.044	7.34	3.788	11.573	8.303
**Blosc,lz4hc,level = 9, shuffle**	1.162	2.471	5.744	2.672	12.636	6.479
**Blosc,lz4hc,level = 9,w/o shuffle**	1	3.285	6.232	2.371	8.477	4.707
**Blosc,lz4,level = 6, bitshuffle**	1.119	2.907	6.424	3.654	10.453	8.019
**Blosc,lz4,level = 6, shuffle**	1.094	1.889	3.185	2.313	7.143	5.243
**Blosc,lz4,level = 6,w/o shuffle**	1	1.639	2.374	1.508	2.872	1.726
**Blosc,lz4,level = 9, bitshuffle**	1.122	2.977	6.683	3.664	10.572	8.05
**Blosc,lz4,level = 9, shuffle**	1.096	1.9	3.196	2.411	7.16	5.508
**Blosc,lz4,level = 9,w/o shuffle**	1	1.638	2.374	1.512	2.872	1.726
**Blosc,zlib,level = 6, bitshuffle**	1.155	3.096	8	3.785	12.524	8.561
**Blosc,zlib,level = 6, shuffle**	1.281	3.461	7.918	3.673	17.007	8.952
**Blosc,zlib,level = 6,w/o shuffle**	1.14	4.339	8.461	3.094	12.958	6.809
**Blosc,zlib,level = 9, bitshuffle**	1.156	3.109	8.144	3.826	12.732	8.613

**Blosc,zlib,level = 9, shuffle**	1.284	3.504	8.416	3.676	17.613	8.954
**Blosc,zlib,level = 9,w/o shuffle**	1.14	4.244	9.353	3.126	13.978	6.989
**Blosc,zstd,level = 6, bitshuffle**	1.155	3.608	11.201	3.887	13.253	8.753
**Blosc,zstd,level = 6, shuffle**	1.279	3.453	8.235	3.841	17.568	9.409
**Blosc,zstd,level = 6,w/o shuffle**	1.121	4.412	8.346	3.291	12.729	7.004
**Blosc,zstd,level = 9, bitshuffle**	1.161	3.63	11.449	3.943	13.547	8.855
**Blosc,zstd,level = 9, shuffle**	1.283	3.871	9.439	3.935	20.276	9.929
**Blosc,zstd,level = 9,w/o shuffle**	1.193	5.022	10.972	3.619	16.87	8.547
**bzip2,level = 6, shuffle**	1.266	3.579	9.368	4.233	20.355	10.472
**bzip2,level = 6,w/o shuffle**	1.227	5.918	12.006	4.209	19.7	10.352
**bzip2,level = 9, shuffle**	1.265	3.643	9.355	4.236	20.405	10.485
**bzip2,level = 9,w/o shuffle**	1.235	5.923	12.029	4.216	19.752	10.377
**gzip,level = 6, shuffle**	1.28	3.448	7.887	3.726	17.214	9.233
**gzip,level = 6,w/o shuffle**	1.14	4.506	8.465	3.182	13.077	6.865
**gzip,level = 9, shuffle**	1.283	3.504	8.439	3.729	18.264	9.243
**gzip,level = 9,w/o shuffle**	1.14	4.408	9.542	3.229	14.427	7.169
**lz4,nbytes = 16384**	1	1	1	1	2.867	1.726
**lz4,nbytes = 2048**	0.998	1.621	2.335	1.474	2.824	1.721
**lzf, shuffle**	1.106	2.028	3.747	2.509	8.051	5.662
**lzf, w/o shuffle**	1	2.429	3.162	2.078	3.951	2.541
**zstd**	1.12	4.41	6.893	3.276	10.546	5.97

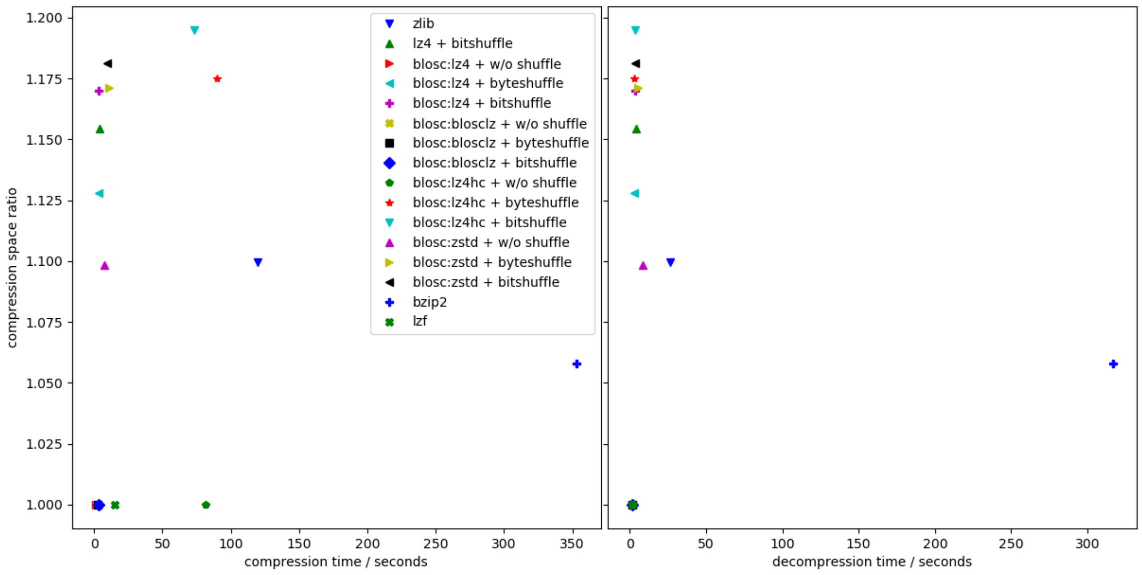

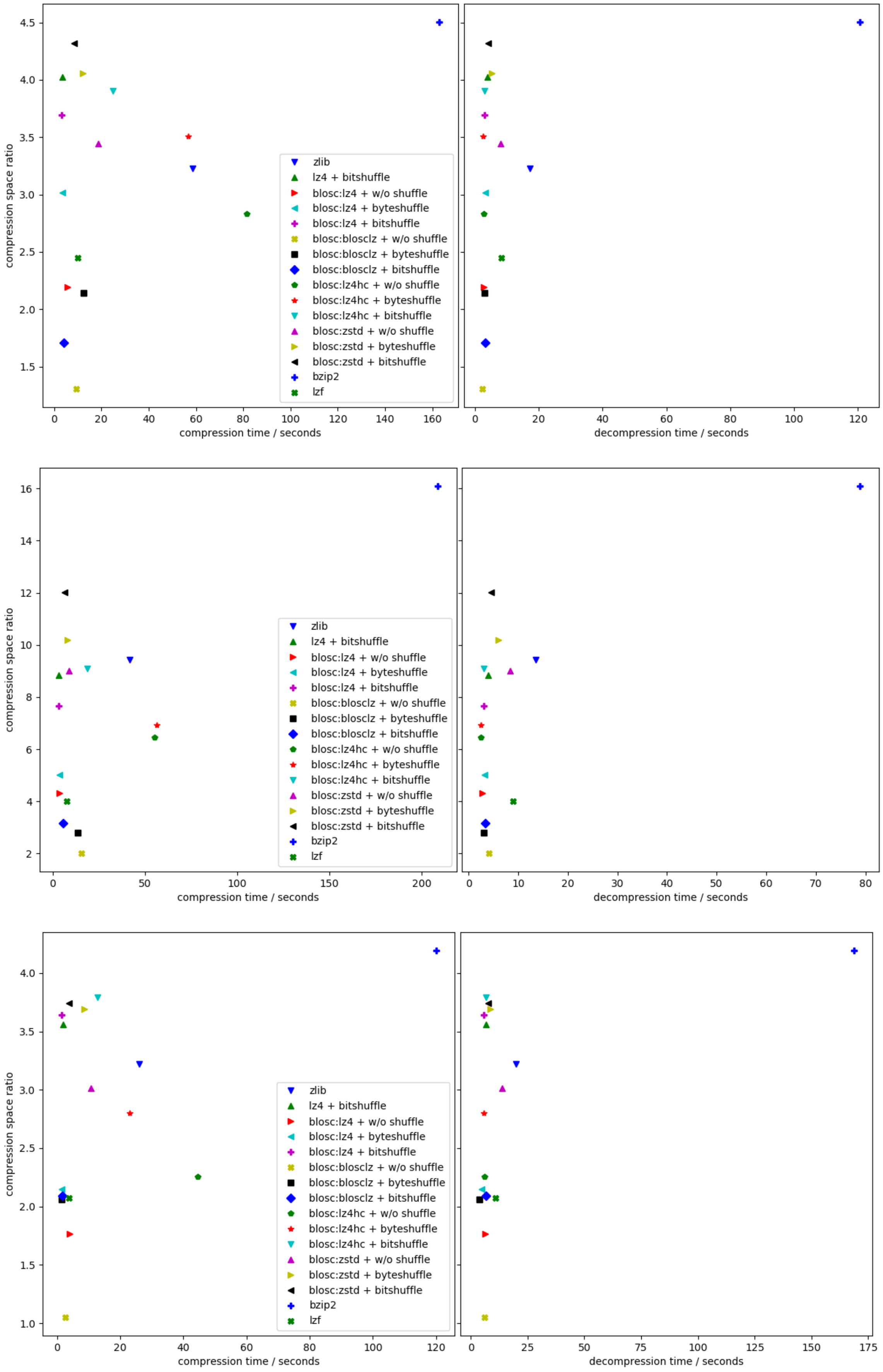
compression space ratio
compression time / seconds
decompression time / seconds
zlib
lz4 + bitshuffle
blosc:lz4 + w/o shuffle
blosc:lz4 + byteshuffle
blosc:lz4 + bitshuffle
blosc:blosclz + w/o shuffle
blosc:blosclz + byteshuffle
blosc:blosclz + bitshuffle
blosc:lz4hc + w/o shuffle
blosc:lz4hc + byteshuffle
blosc:lz4hc + bitshuffle
blosc:zstd + w/o shuffle
blosc:zstd + byteshuffle
blosc:zstd + bitshuffle
bzip2
lzf

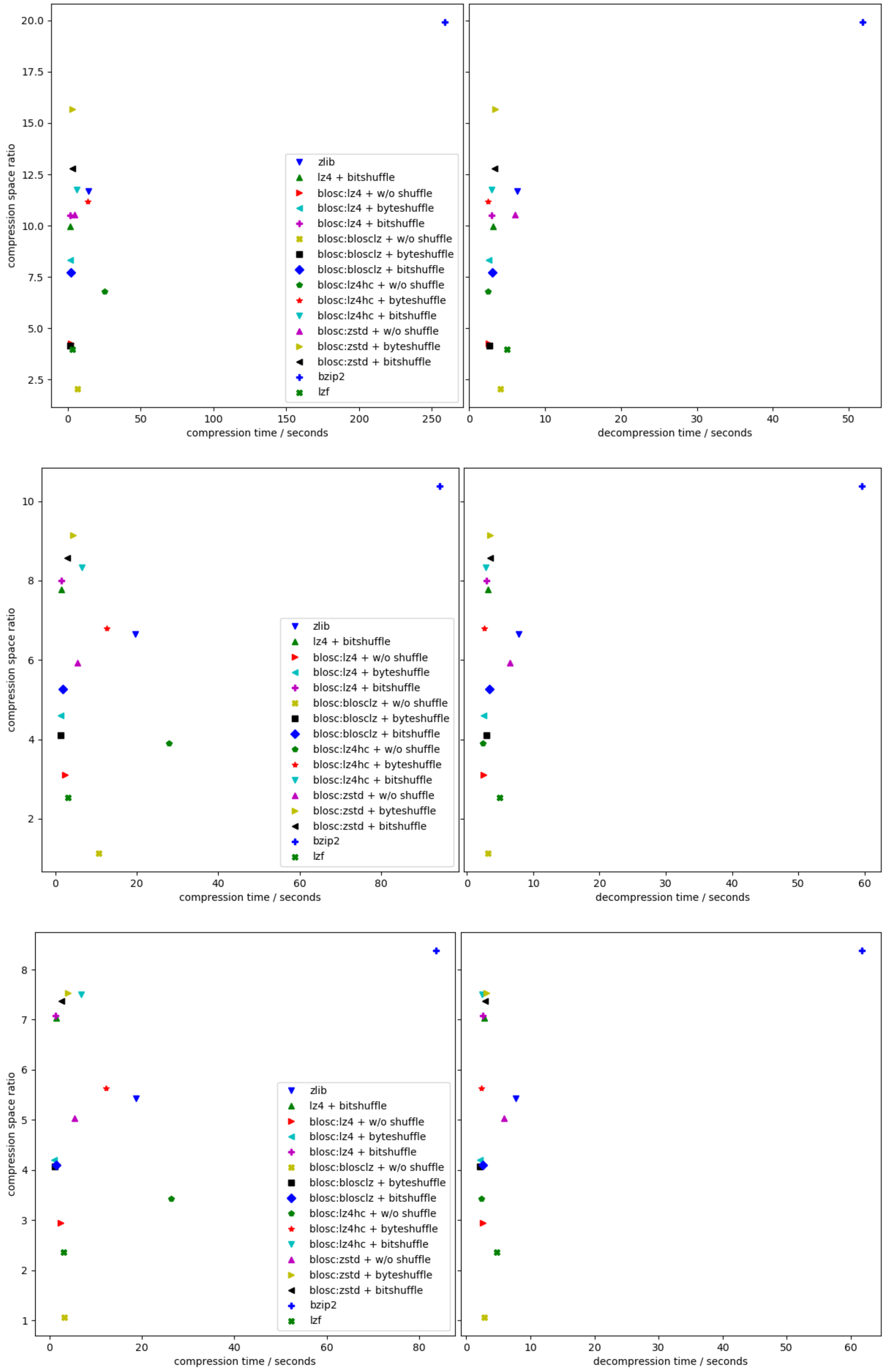
compression space ratio
compression time / seconds
decompression time / seconds
zlib
lz4 + bitshuffle
blosc:lz4 + w/o shuffle
blosc:lz4 + byteshuffle
blosc:lz4 + bitshuffle
blosc:blosclz + w/o shuffle
blosc:blosclz + byteshuffle
blosc:blosclz + bitshuffle
blosc:lz4hc + w/o shuffle
blosc:lz4hc + byteshuffle
blosc:lz4hc + bitshuffle
blosc:zstd + w/o shuffle
blosc:zstd + byteshuffle
blosc:zstd + bitshuffle
bzip2
lzf

Figure S1 Relationship between compression ratio and compression/decompression speed for 1000 diffraction patterns obtained from various detectors, including data subjected to lossy data reduction. From top to bottom: AGIPD, lysozyme, floating-point data; AGIPD, lysozyme, integer data; AGIPD, lysozyme, integer truncated to 1 bit; Eiger 2X 16M, lactamase; Eiger 2X 16M, lactamase, truncated to 1 bit; Eiger 2X 16M, lactamase, truncated to 3 bits; Eiger 2X 16M, only patterns with many photons.

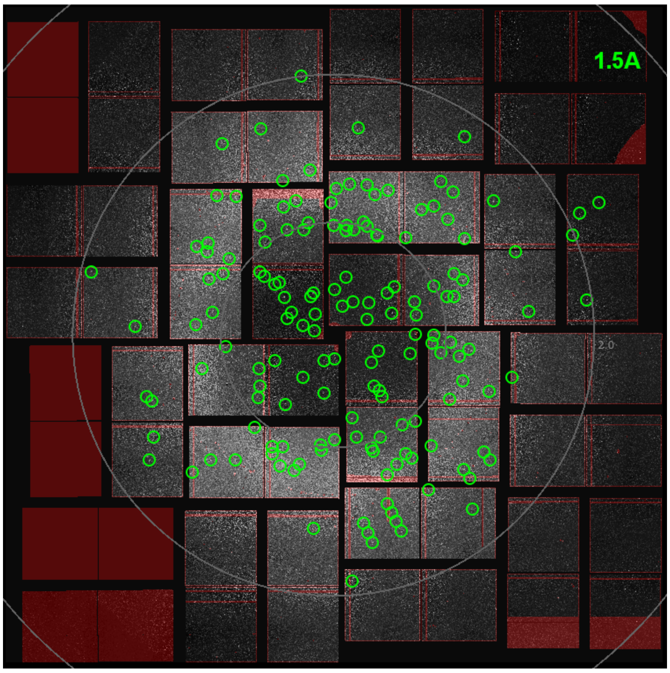

Figure S2 Diffraction pattern of lysozyme measured with the CS-PAD detector. Red regions were masked and not considered during the data processing. Green circles indicate the found peaks. The resolution rings demonstrate the fact that there is almost no data measured below 1.5A resolution.

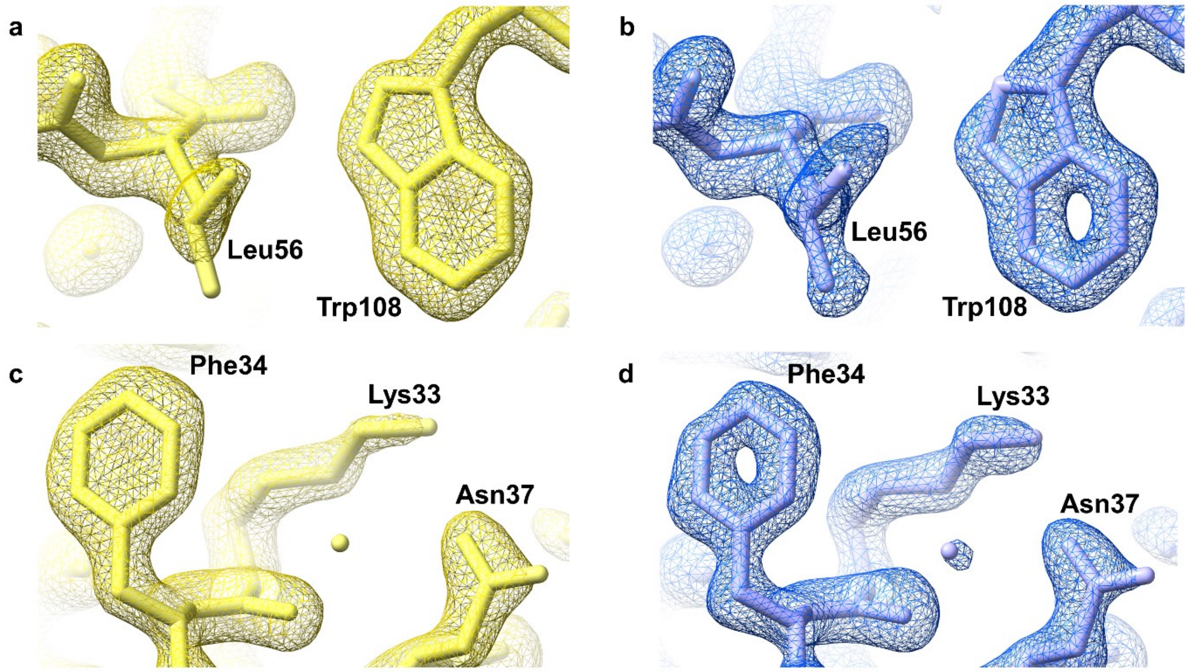

Figure S3 Additional examples of improved electron density comparing original (yellow, a/c) and re-processed data (blue, b/d). All maps are contoured at σ = 1.5. (a/b) Residues Leu56 is located in the core of the protein and Trp108 is located within the active site cleft. (c/d) Residues Phe33, Lys34 and Asn37 are located at the surface of the protein.

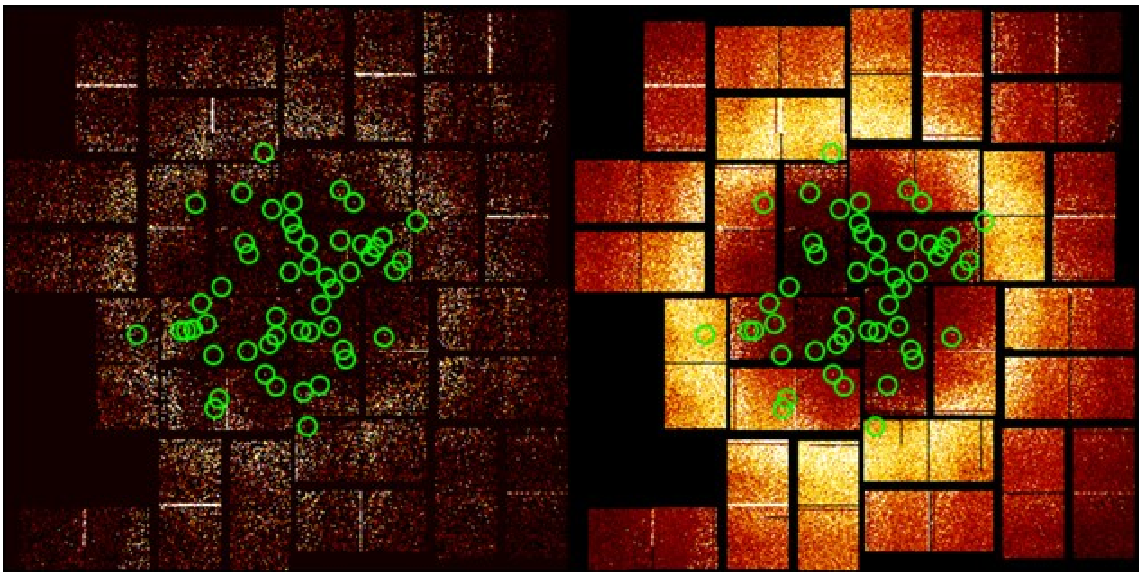

Figure S4 The same diffraction pattern processed in 2011 using local background subtraction (left) and re-processed recently (right). Green circles mark the determined Bragg peaks.

Table S3 Comparison Phenix/1.20 versus Phenix/1.13 refinement results (using default parameters, without any manual interventions).

**Resolution range**	**R_{free}/R_{work}, Phenix/1.20**	**R_{free}/R_{work}, Phenix/1.13**
20 Å - 1.9 Å	0.205/0.171	0.201/0.165
20 Å - 1.5 Å	0.216/0.198	0.204/0.188

Table S4 The results of applied non-hits rejection approaches (with/without 2x2 binning) on data collected for different samples (ferritin, lysozyme, MPro, lactamase) in November 2020 at P11, Petra III with Eiger 2X 16M detector

**Subset of sample**	**Raw**			**Raw, only hits**				**Binned, only hits**			
	**Vol.**	**Num. patterns/hits**	**Indexed patterns/crystals**	**Vol.**	**Num. patterns/hits**	**Indexed patterns/crystals**	**CR**	**Vol.**	**Num. patterns/hits**	**Indexed patterns/crystals**	**CR**
Ferritin	8.66T	1025989 /6808	6500 /7010	134.2 G	15254 /6620	6237 /6237	66.08	84.2G	31929 /10589	7280 /7762	105.32
Lysozyme (1)	3.7T	374000 /62741	42881 /61893	721G	75829 /62031	42564 /61341	5.25	310G	97874 /60622	42781 /62028	12.22
MPro	3.7T	400000 /8978	5389 /5648	192G	20951 /13764	8438 /8927	19.73	42G	11883 /11883	8629 /9407	90.21
Lysozyme (2)	260G	40000 /23151	5809 /6476	193G	30651 /27791	7888 /9055	1.35	85G	33823 /29146	9238 /10820	3.06
Lactamase	1.9T	200000 /198181	187708 /507045	1.8T	199606 /198088	187826 /505330	1.06	711G	199779 /199779	188919 /554576	2.74

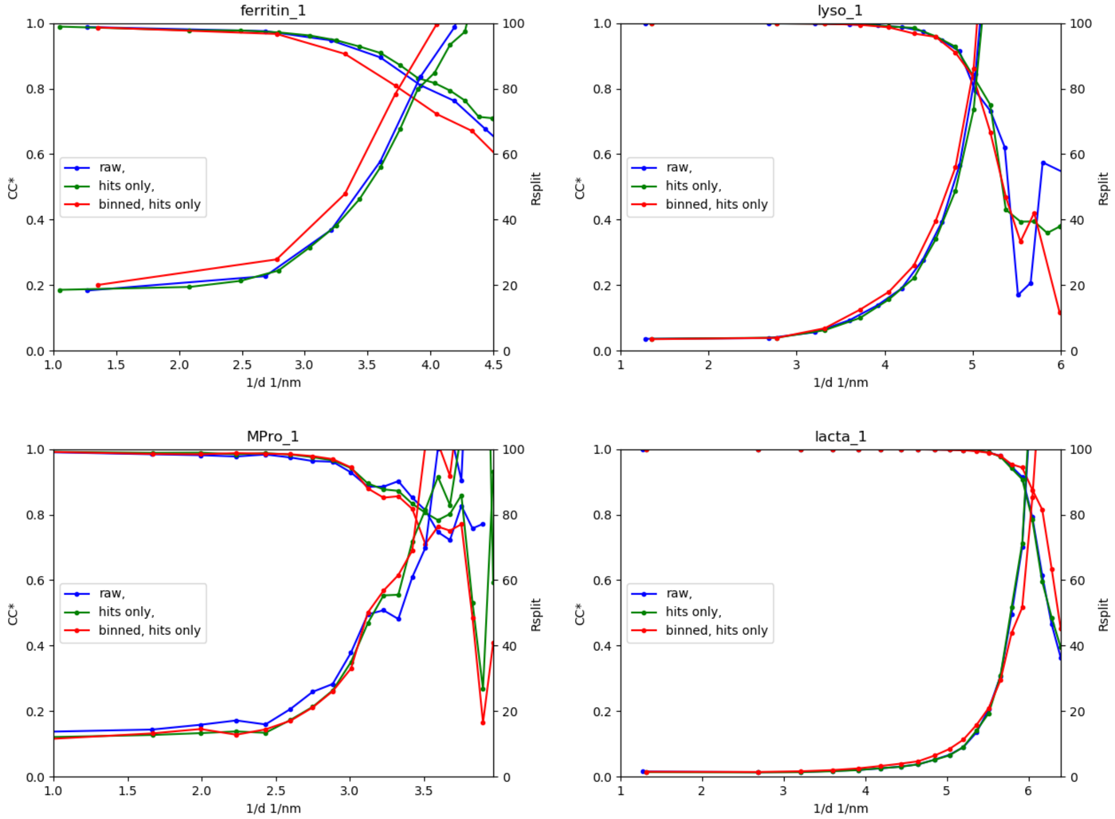

Figure S5 CC* and R_{split} metrics for four different samples: ferritin, lysozyme, MPro, and lactamase. Blue curves correspond to the processing of only raw data, green – only patterns with determined crystal diffraction (hits), and the red curve – binned hits.

Table S5 The result of a quantization approach with constant steps performed on the AGIPD lysozyme dataset (in this case 1 photon was equal to 73 ADUs), consisting of only hits. The data from the detector is calibrated and usually saved as "native float" (so-called "processed data").

	**Number of patterns**	**Indexed patterns/crystals**	**R_{free}/R_{work}**	**CR for gzip without shuffle**	**CR for gzip with shuffle**
float (original)	189960	166841/236099	0.1670/0.1497	1.102	-
int	189960	166840/236117	0.1689/0.1501	3.25	4.105
rounded to 16 ADUs	189960	166837/236145	0.1666/0.1499	-	5.926
rounded to 64 ADUs	189960	166811/236182	0.1677/0.1504	-	8.869
rounded to 256 ADUs	189960	166800/236215	0.1690/0.1509	-	21.167
rounded to 1024 ADUs	189960	166774/235873	0.1753/0.1543	46.829	63.578
rounded to 4096 ADUs	189960	159441/225858	0.2431/0.1993	-	650.586

Table S6 Results of influence of two lossy compression schemes: rounding to 64 and 1024 ADUs and using less data (1/16 of the original dataset). Diffraction from lysozyme crystals measure at eX-FEL using AGIPD.

Part	Num. patterns/hits	Indexed patterns/crystals	R_{free}/R_{work}
int (all)	189960/189960	166840/236117	0.1689/0.1501
int (1/16)	11873/11873	10463/14679	0.1846/0.1625
rounded to 64 ADUs (all)	189960/189960	166811/236182	0.1677/0.1504
rounded to 64 ADUs (1/16)	11873/11873	10423/14720	0.1881/0.1619
rounded to 1024 ADUs (all)	189960/189960	166774/235873	0.1753/0.1543
rounded to 1024 ADUs (1/16)	11873/11873	10408/14726	0.1856/0.1618

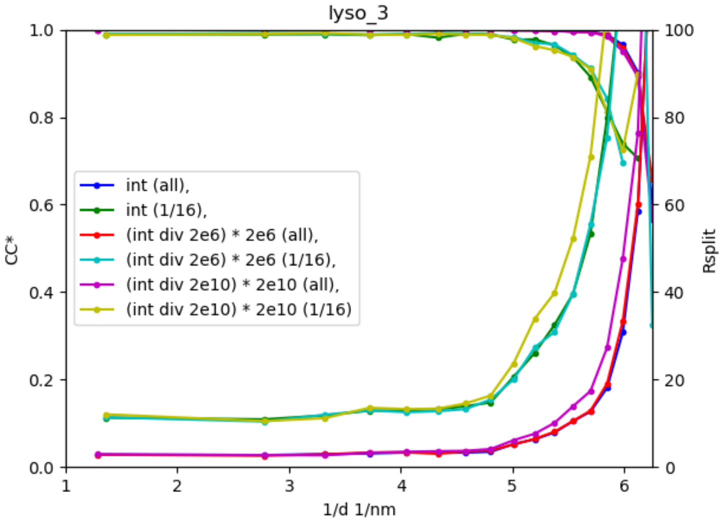

Figure S6 Data quality (CC* and R_{split}) for the datasets rounded to 1024 ADUs and for a small sub-set (1/16) of the same data. Diffraction from lysozyme crystals measure at eXFEL using AGIPD.

Table S7 Examples of the rounding integer values to three most significant bits including the floating-point representation. The bits that are saved are marked in green and the bit responsible for the rounding – in yellow. The code is deposited on GitHub (https://github.com/galchenm/binningANDcompression.git)

Initial number	Binary representation of the initial number	Binary representation of the resulting number	8-bit floating-like representation	Resulting number
81	0101 0001	0101 0000	00011101	80
87	0101 0111	0101 0000	00011101	80
88	0101 1000	0110 0000	00011110	96
258	0001 0000 0010	0001 0000 0000	00100100	256

1316	0101 0010 0100	0101 0000 0000	00101101	1280
1450	0101 1010 1010	0110 0000 0000	00101110	1536

Table S8 Influence of different quantization lossy compression on data (lysozyme, AGIPD) quality. The data was rounded to 64 and 73 (one photon) ADUs and also leaving only 3 and 1 the most significant bits was tested.

Type	Num. patterns /hits	Indexed patterns /indexed crystals	R_{free}/R_{work} (10 Å - 1.69 Å)	CR, gzip + shuffle	CR, bzip2 + shuffle
Int	82798/82798	34720/34821	0.2048/0.1653	3.946	3.577
Rounding to 64 ADUs	82798/82798	34715/34830	0.2072/0.1632	5.137	5.926
Photon conversion (to 73 ADUs)	82798/82798	34685/34801	0.2077/0.1680	5.319	5.855
Rounding to 3 bits	82798/82798	34698/34812	0.2065/0.1655	5.421	6.124
Rounding to 1 bit	82798/82798	34447/34565	0.2048/0.1663	8.751	10.280

Table S9 Overall statistics for SAD dataset of thaumatin (measured at SwissFEL with JUNGFRAU 16M detector): original and reduced in different ways.

	raw	binned	binned, 3 bits	binned, 2 bits	binned, 1 bit
Num. patterns/Num. hits	52207/52207	52207/51906	52207/51784	52207/51597	52207/52184
Indexed patterns/ Indexed crystals	50844/59004	47929/53635	47499/53040	46221/51264	26965/28171
Volume (GB)	3300	681	105	87	69
Volume 8 bits “float” (GB)	-	-	93	75	57
Resolution (Å)	25.78 - 2.42	25.78 - 2.42	25.78 - 2.42	25.78 - 2.42	25.78 - 2.42
R_{split} (%)	5.97	6.35	6.65	6.81	7.94
CC1/2	0.993	0.994	0.993	0.992	0.991
CC*	0.998	0.998	0.998	0.998	0.998
CCano	0.327	0.320	0.247	0.271	0.251
SNR	16.00	14.71	13.52	13.25	10.53
Completeness (%)	89.79	88.14	88.59	88.26	88.77
Multiplicity	287.50	251.60	276.58	264.28	172.36
Total Measurements	4952834	4254810	4701093	4474988	2935670
Unique Reflections	17227	16911	16997	16933	17032
Wilson B-factor (Å^2)	132.16	194.1	157.77	111.7	133.63

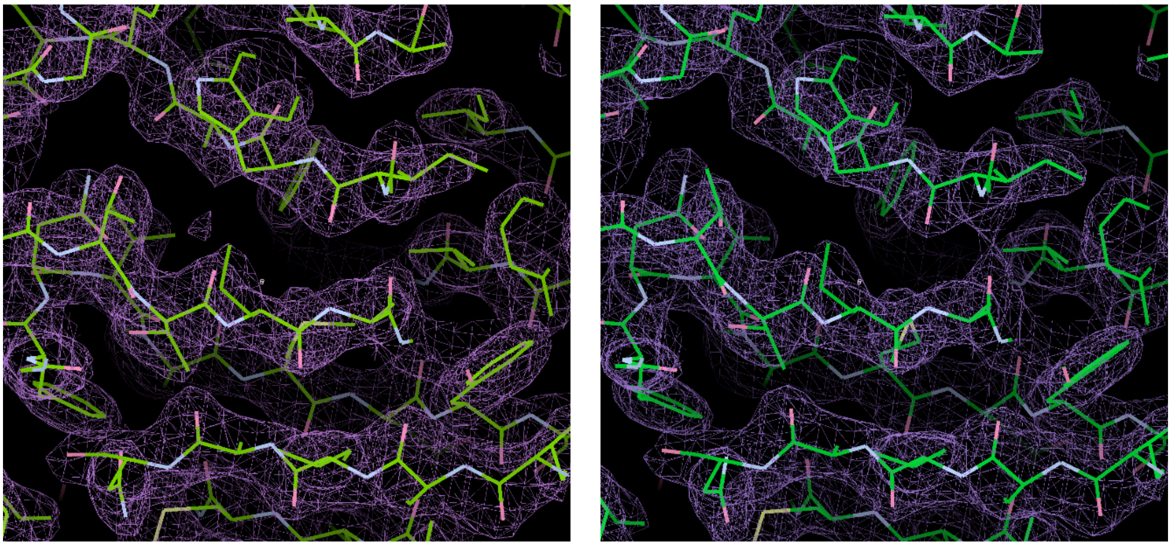

Figure S7 The structure and weighted difference electron density map (2Fo-Fc) made with Autobuild for the Thaumatin SAD data: left – binned data, right – binned data rounded to the 3 most significant bits.
